# Fear of Illness and Convenient Access to Vaccines Appear to Be the Missing Keys to Successful Vaccination Campaigns: Analysis of the Factors Influencing the Decisions of Hospital Staff in Poland concerning Vaccination against Influenza and COVID-19

**DOI:** 10.3390/vaccines10071026

**Published:** 2022-06-27

**Authors:** Robert Susło, Piotr Pobrotyn, Artur Mierzecki, Jarosław Drobnik

**Affiliations:** 1 Epidemiology and Medical Education Unit, Population Health Department, Health Sciences Faculty, Wrocław Medical University, 50-556 Wrocław, Poland; jaroslaw.drobnik@umw.edu.pl; 2 Citodent, 50-403 Wrocław, Poland; piotr@citodent.pl; 3 Family Medicine Department, General Medicine and Dentistry Faculty, Medical University in Szczecin, 70-203 Szczecin, Poland; artur.mierzecki@pum.edu.pl

**Keywords:** vaccination, human influenza, COVID-19, hospital staff, vaccination refusal, vaccination hesitancy, immunization programs, persuasive communication

## Abstract

The COVID-19 pandemic has lasted for two years as of 2022, and it is common knowledge that vaccines are an essential tool to mitigate the health, economic, and social fallout. Unfortunately, vaccine hesitancy is still a serious global problem, both in the general population and among healthcare workers. The authors used an original questionnaire to conduct an anonymous survey study in the University Clinical Hospital in Wrocław, Poland, in April and May of 2021 after acquiring consent from the Medical University of Wrocław, Poland Bioethical Committee. The study results demonstrate that, to a significant extent, the decisions concerning vaccinations are based on factors that are difficult to change with rational argumentation, including people’s personal opinions or beliefs concerning vaccinations and their earlier experiences with vaccinations. The study results suggest that the impregnating effect of one’s own opinions, beliefs, and experiences can be surmounted if vaccines are dispensed free and conveniently while the pathogen is irrationally and emotionally perceived as untamed and possibly severe and life threatening. It makes a significant difference as in such cases that the percentage of participants whose decisions concerning vaccination are influenced by the risks to life or health of one’s own or others rises by about 27 and 36 percent points, respectively. Therefore, in order to succeed, campaigns for vaccinations need to include strong subjective and emotional communication, appealing to negative emotions and exploiting the public’s fear of the unknown while stressing tangible and personal threats possibly resulting from acquiring a vaccine-preventable infectious disease.

## 1. Introduction

The COVID-19 pandemic has been ongoing for two years as of 2022. It is common knowledge that vaccines are an important tool to mitigate the health, economic, and social fallout of the COVID-19 pandemic [1]. The COVID-19 vaccination limits the risks of SARS-CoV-2 infection, reducing its symptoms and severity [2], as well as the otherwise significant risk of transmission to other people. In the case of healthcare workers, it includes other staff members, patients [3], and residents [4]; in this way, the COVID-19 vaccination also helps keep healthcare services uninterrupted [5]. It is widely agreed that hospital staff members belong to the priority groups crucial for the success of the COVID-19 vaccination scheme in Europe [6] and the USA [7]. Several COVID-19 vaccines have been available for over a year now, but it is crucial for an effective vaccination campaign that people are not only able but also willing to get vaccinated as soon as possible [1].

Vaccine hesitancy is still a serious global problem in the general population. The attitude toward the COVID-19 vaccine tends to be worse than the acceptance level of seasonal influenza vaccines, which is still very limited even though it has been available for many years [5]. However, medical staff members surveyed in different countries present, on average, high levels of willingness to get the COVID-19 vaccine—ranging from 51% in Greece [8], 55% in Russia [9], 63% in the USA [7], and 76% in Vietnam [10] to ~90% in China [9] and even 95% in Thailand [11]. The vast majority are also willing to suggest vaccination to their family members (~78%) and patients (~81%) [11]. The positive attitude toward vaccination among healthcare workers (HCWs) is especially important, as they may be considered role models or trendsetters for the general population; there is an association between vaccination coverage in HCWs and the general local population [12,13].

Before the introduction of vaccines, the observed SARS-CoV-2 transmission was high, especially among healthcare workers worldwide [14]. This was facilitated by the fact that the symptoms of COVID-19 were highly uncharacteristic, often mild or even absent [15]. In fact, approximately half of the infected hospital staff members in whom anti-S antibodies were detected did not experience any COVID-19 symptoms, thus presenting as silent infection spreaders. Considering the above factors, the refusal to get vaccinated presents a serious issue that prevents improvement of the situation [14]. As in the pandemic crisis, limited acceptance levels are not likely to be considered satisfactory in the context of patients’ health safety. In the USA, it was affirmed that healthcare employers have the legal right to require COVID-19 vaccination from their staff [16], despite the ethical dilemmas involved [17].

Among the factors increasing vaccine acceptance are trust in the vaccine and the belief that it is effective [9]; the perceived susceptibility and known severity of COVID-19 [10]; high level of vaccine literacy [7]; the will to prevent the spread of the disease [8,9]; the will to protect family members [8] or individuals with weak immune systems; the fact that everyone else around them is already vaccinated and being male and of older age [6,7,8,9,18,19]; high levels of confidence, having the feeling of a collective responsibility [9]; worrying about COVID-19 and being confident about the way the epidemic is being managed [19]; a higher level of education [7] (doctoral or Master’s degree, higher educational levels, or basic science laboratory workers) [20], especially being a physician [6,8,18]; treating vaccination as a means to reduce social isolation [21]; reported influenza vaccination [6,22] of at least once during the last three seasons [8,18] or complete vaccination against hepatitis B [8]; in addition to having the impression of being privileged to receive the vaccine [23].

In contrast, the factors reported as increasing vaccine hesitancy are a low level of vaccine literacy [8,24]; uncertainty of the efficacy of the vaccine, and fear of adverse effects [6,7,8,19,20,24,25], including a possible negative impact on pregnancy [26,27]; the COVID-19 vaccine’s newness [7]; distrust of the vaccine’s contents [20] or the pharmaceutical industry [19]; lack of choice of the preferred kind of vaccine [11]; being female [24,25,27]; being young [24,25,27] (especially <50 years of age) [20]; belonging to a non-White ethnic group [7,25,27]; being of general good health, and working without known contact with COVID-19 patients [24]; being a non-doctor healthcare employee (nurse/midwife, administrative personnel, patient attendant/cleaning staff, or security guard) [13,19,20,24,25]; having a history of confirmed SARS-CoV-2 infection in the past [20,27]; having rejected the influenza vaccine [8,20,25]; hesitancy about other vaccines [20]; transportation obstacles making it difficult to reach a vaccination point; and social vulnerability due to housing conditions [13].

It is worth stressing that declared willingness to vaccinate is known to not always fully translate into actual vaccinations; the highest coherence level in this respect is typical for physicians [18].

The Internet and social media, together with direct interpersonal communication, are considered important sources of influence, both positive and negative, on people’s perceptions and willingness to receive COVID-19 vaccination [28]. A common role in the decision-making process is receiving information from relatives [10].

It is worth mentioning that, on average, ~75% of people vaccinated against COVID-19 consider their tolerance of the vaccination as good after the first dose and 50% after the second dose. Side effects following COVID-19 vaccination are relatively common but, in most cases, mild—including pain at the injection site, fatigue, headaches, and myalgia—which manifest themselves with decreasing frequency. They cause only a temporary inability to work and only in a limited proportion of vaccinated staff—after the first dose in ~2% of workers and after the second dose in ~20% of workers [29].

In Poland, data concerning the COVID-19 vaccination is treated as private, secret, and sensitive. Consequently, the current laws do not give anyone the right to demand information concerning COVID-19 vaccination status from citizens, including workers, without any exceptions (also for healthcare workers). However, such information can be made available by the person in order to be exempted from the limitations imposed on the public of unknown COVID-19 vaccination status, i.e., they are not counted in the maximum number of people allowed to enter and stay at certain locations [30].

Based on the pooled data retrieved from the state registry of the administered vaccination doses, a significant part of the Polish public has denied vaccination. However, according to the official statistics, COVID-19 vaccination is popular enough among healthcare workers in Poland. Additionally, independent estimations were made by organizations, including the self-governments of particular health professions. The medical staff in Poland were vaccinated immediately after the first COVID-19 vaccine was registered in the European Union on 21 December 2020; thus, they were predominantly administered a two-dose course of the Pfizer COMIRNATY COVID-19 vaccine, which was the first available on the market [31]. The introduction of the COVID-19 vaccine in Poland was eagerly expected, and, in the beginning, the vaccination received a warm welcome mixed with high levels of interest in society [32]. The Self-Government of the Polish Physicians and Dentists announced, in March 2021, that 64,804 physicians (46% of all practicing in Poland) and 15,392 dentists (40% of all practicing in Poland) underwent COVID-19 vaccination. This information is inconsistent with the information that was shared one month earlier by the Polish Ministry of Health, which claimed that 90% of Polish physicians had been vaccinated against COVID-19 at that time [33].

The Ministry of Health shared similar information in July 2021 again, this time providing the exact numbers of the Polish healthcare workforce vaccinated against COVID-19. According to that data, among the 157,197 physicians practicing at that time, 146,147 (92.97%) had accepted the first dose of the COVID-19 vaccine, and 142,532 (90.67%) completed the full vaccination scheme (accepted the second dose of the Pfizer COMIRNATY vaccine). Among the 44,230 active Polish dentists, in July 2021, there were 39,881 (90.17%) who had accepted the first dose of the COVID-19 vaccine and 37,748 (85.34%) were already fully vaccinated. Out of the 300,510 professionally active nurses at that time, 268,803 (89.45%) were vaccinated with the first dose of the COVID-19 vaccine, and 242,756 (80.78%) had completed the full course of vaccination. Among the 39,309 midwives working in Poland in July 2021, 35,435 (90.14%) were vaccinated with one dose of the COVID-19 vaccine, and 31,679 (80.59%) accepted the full course of vaccination. In addition, pharmacists accepted the COVID-19 vaccine often: 34,603 (88.43% of the total number of the 39,131 professionally active at that time in Poland) accepted the first dose of the vaccine and 31,419 (80.29%) of them were fully vaccinated [34]. There are, so far, no data available by the Ministry of Health concerning the COVID-19 vaccine acceptance among staff other than medical profession members employed at medical facilities, involved in patient care or rehabilitation, but also in cleaning, technical maintenance, administrative or security staff. Meanwhile, studies have demonstrated that patient support staff and facilities and administrative personnel are less willing than HCWs to participate in activities preventing the possible spread of infection [35].

The data concerning the high level of acceptance of the COVID-19 vaccination among Polish medical staff contrast sharply with the data concerning the Polish general public. Based on the pooled data retrieved from the state registry of the administered vaccination doses, a significant proportion of the Polish public has denied vaccination. According to the Ministry of Health, up to 13 December 2021, there were 44,200,031 doses of COVID-19 vaccine administered in Poland and 20,685,210 people who were subjected to the full course of the given COVID-19 vaccine required to be considered fully vaccinated [36].

Vaccinating the largest part of the society and reaching the level of “herd immunity” to SARS-CoV-2 infections is a crucial element of the strategy for ending the COVID-19 pandemic. Therefore, it is vital to investigate the factors that are considered most often in the decision process, as well as the possible differences demanded between the vaccination campaign targeting in the case of COVID-19 and other infectious diseases.

## 2. Materials and Methods

The authors carried out an anonymous survey study in the University Clinical Hospital in Wrocław, Poland, in April and May 2021 after acquiring consent from the Medical University of Wrocław, Poland Bioethical Committee, and the Management of the University Clinical Hospital. The hospital is a 1677-bed facility and employs 4998 staff members, including 1554 doctors, 1478 nurses, 1085 other members of medical personnel and 305 administrative workers and 576 members of other non-medical personnel.

The authors compiled the original questionnaire after an extensive literature review on the topic and approached hospital staff members directly to ask open questions about attitudes toward vaccinations, with special stress on COVID-19 vaccinations. The questionnaire was divided into a general section and a detailed section. The general section of the questionnaire included questions concerning the characteristics of the respondents, including age and closed questions about sex, occupation, place of living, and history of previously accepted vaccinations against influenza and COVID-19. The detailed section of the questionnaire contained the closed questions concerning the factors taken into account by respondents before they made their decision about vaccination against influenza and against COVID-19, including the health status of the person at the time the vaccination; subjective judgement of the risk of getting ill because of infection; the subjective judgement of the risk of serious complications or death because of infection; the subjective judgement of the risk of passing the acquired infection to other people; the availability of the vaccine; the cost of vaccination; the opinions concerning vaccination found in professional medical information sources, popular journals, and the press or on the radio, television programs, the Internet, or social media; the opinions concerning vaccination expressed by clergy members, renowned philosophers, acquaintances, relatives, or family; the behavior or example set by acquaintances or family; the personal opinions or beliefs of the respondent concerning vaccinations; the prior experiences of the respondent with vaccinations; the last, open question concerned other influencing factors. Respondents also had the possibility of expressing their objection against answering all of the questions contained in the questionnaire.

The authors distributed 700 paper questionnaires among all the hospital staff willingly claiming to be vaccinated against COVID-19 at the time of the study, out of which 659 were returned. The participants were included in the study based on the following criteria: presence and performing any work at the hospital during the time of the study (excluding staff who were on sick leave or vacation) and willingly agreeing to participate in the survey. The participants answered the questionnaires independently and anonymously, but assistance, explanations and additional information was provided to them on request. The questionnaire results were transferred into an Excel 2013 spreadsheet (Microsoft, Redmond, WA, USA) and subjected to statistical analysis using the data analysis software system Statistica 13.3 PL (2017, TIBCO Software Inc., Palo Alto, CA, USA). The quantitative study used contingency tables along with the chi-square test, chi-square maximum likelihood or chi-square with Fisher’s correction applied appropriately to the calculated expected observations. Basic descriptive statistics were calculated for the tested quantitative values for the entire material as well as for the assumed test groups. The threshold for the statistical significance of differences between groups was set at *p* < 0.05.

## 3. Results

The returned questionnaires were completed by 540 (81.94%) of the participants who declared themselves as women, 109 (16.54%) as male, and 10 (1.52%) participants who did not declare themselves as belonging to any sex.

**Figure 1:**
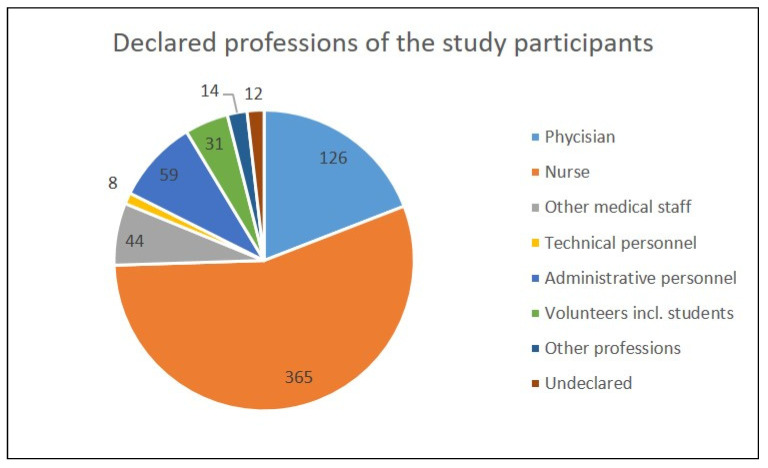
Number of study participants divided into groups based on their declared professions.

Most of the study participants declared that they worked as nurses (55.39%). The others included physicians (19.12%), other medical personnel (6.68%), members of administrative (8.95%) or technical (1.21%) staff, volunteers, including medical students, supporting the regular medical staff during the COVID-19 pandemic (4.70%), other professions (2.12%), or an undeclared occupation (1.82%)—as shown in Figure 1.

All of the study participants were vaccinated against COVID-19. Among the study participants, there were 350 (53.11%) who were not vaccinated against influenza during the previous season, 282 (42.79%) who accepted the vaccine, and 27 (4.10%) that did not declare any vaccination status.

The place of residence of the respondents varied considerably: 460 (63.13%) of them lived in cities (>250 K inhabitants); 26 (3.95%) lived in big towns (50 K–250 K inhabitants); 78 (11.84%) lived in small towns (up to 50 K inhabitants); 108 (16.39%) lived in rural areas, while 31 (4.70%) did not declare where they lived.

**Figure 2:**
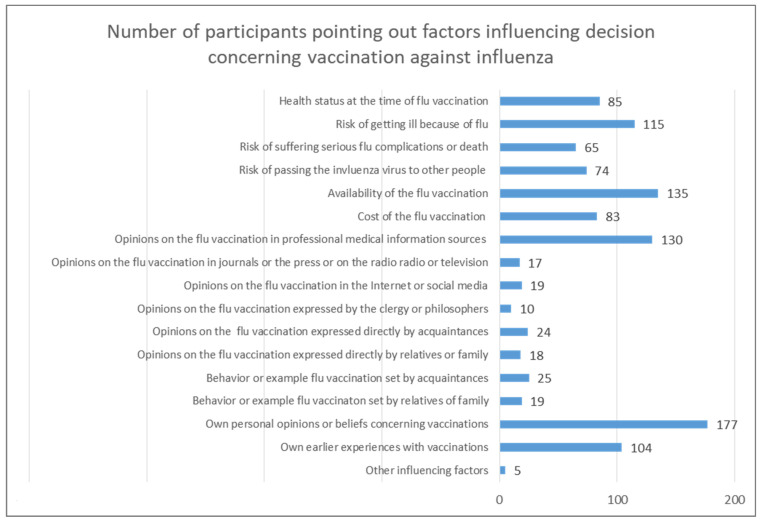
Number of participants pointing out factors influencing their decision concerning vaccination against influenza.

Out of the 659 study participants, 300 (45.52%) respondents decided to share their opinions concerning the factors influencing their decision to vaccinate against influenza (Figure 2). Among the most commonly considered decision factors were the respondent’s personal opinions or beliefs concerning vaccination (59.00%); availability of the flu vaccination (45.00%); opinions concerning the flu vaccination found in professional medical information sources (43.33%); their own subjective judgment of the risk of getting ill from influenza virus infection (38.33%); and their own earlier experiences with vaccinations (34.67%). Significantly less often, the respondents considered in their decision making regarding the influenza vaccination their health status at the time that the flu vaccinations were carried out (28.33%), the cost of the flu vaccination (27.67%), and their own subjective judgment of the risk of passing on the influenza virus to other people (24.67%) or to suffer serious complications or death because of the influenza virus (21.67%). The least popular reasons for the decision to vaccinate were behavior or example set by acquaintances (8.33%) or opinions concerning the flu vaccination expressed directly by them (8.00%); opinions concerning the flu vaccination found on the Internet or social media (6.33%); the behavior or example set by relatives or family (6.33%) or the opinions expressed directly by them (6.00%) found in popular journals or press or on the radio or television programs (5.67%), or expressed by clergy members or renowned philosophers (3.33%). Other influencing factors (1.67%) included perceived pressure from superiors in the workplace.

**Figure 3:**
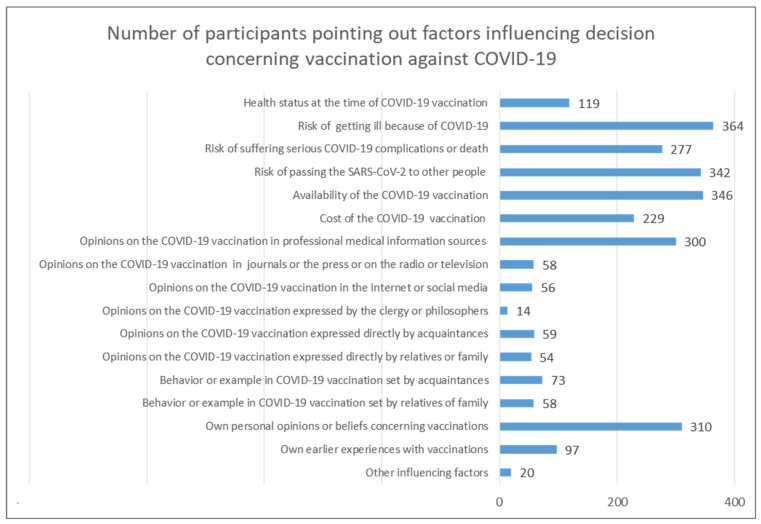
Number of participants pointing out factors influencing their decision concerning vaccination against COVID-19.

There were 560 (85%) respondents who answered the part of the questionnaire investigating the structure of the factors influencing the decisions of the hospital staff members concerning vaccination against COVID-19 (Figure 3). Among the most commonly considered decision factors were their own subjective judgment of the risk of getting ill from SARS-CoV-2 infection (65.00%), the availability of the COVID-19 vaccine (61.79%), and their own subjective judgment of the risk of passing the acquired SARS-CoV-2 infection on to other people (61.07%). Less often, the respondents considered as a basis of their decision for accepting COVID-19 vaccination their own opinions or beliefs concerning vaccinations (55.36%), opinions concerning the COVID-19 vaccination found in professional medical information sources (53.57%), their own subjective judgment of the risk of suffering serious complications or death because of SARS-CoV-2 (49.46%), and the cost of COVID-19 vaccination (40.89%). Somewhat often, the respondents took into account their own health status at the time that the COVID-19 vaccination was carried out (21.25%) and their own earlier experiences with vaccinations (17.32%).

The least popular reasons for the decision to vaccinate against COVID-19 were the behavior or example set by acquaintances (13.04%) or relatives or family (10.36%) and the opinions expressed directly by acquaintances (10.54%), relatives or family (9.64%), clergy members or renowned philosophers (2.50%), in popular journals or the press or on the radio or television programs (10.36%), on the Internet or social media (10.00%), or other influencing factors (3.57%). Among the latter were factors such as perceived pressure from superiors and professional standards in the workplace (mentioned by two people); fear of possible administration-related or legal problems (one person); peer pressure (one person); medical profession-related infection risk (four people); episode of being ill with COVID-19 (one person); needing to stay in safe contact with family (one person); experiencing the death of a family member from COVID-19 (one person); wanting to get rid of COVID-19-related limitations (one person); improving travel safety (one person); and their own observations (one person).

**Figure 4:**
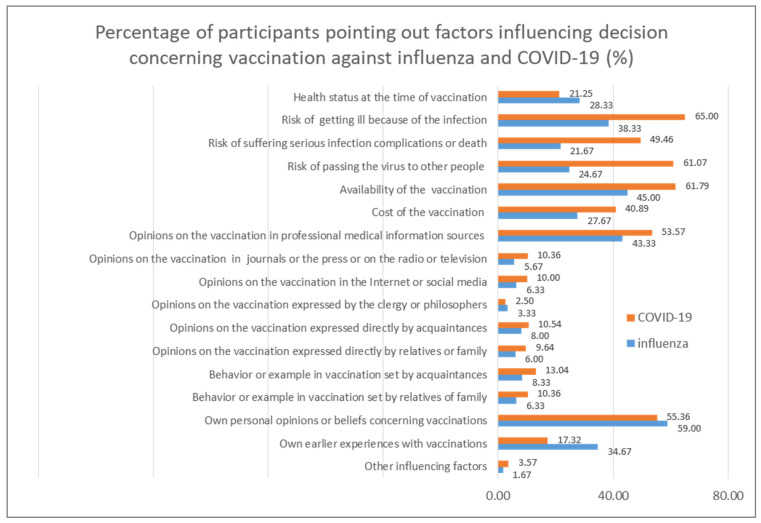
Percentage of participants pointing out factors influencing their decision concerning vaccination against influenza and COVID-19 (%).

A comparison of the percentages of the respondents who pointed out the respective factors influencing their decision to vaccinate themselves against COVID-19 and influenza demonstrated significant differences, as visualized in Figure 4.

**Figure 5:**
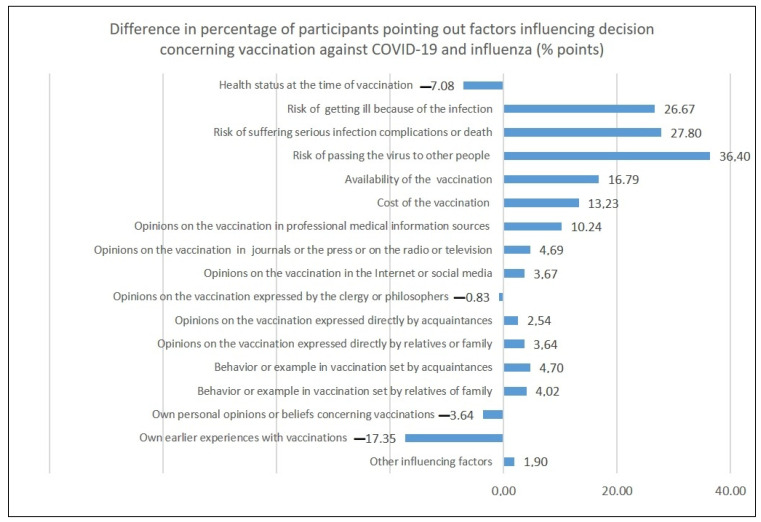
Differences in the percentage of participants pointing out factors influencing their decision concerning vaccination against COVID-19 and influenza (% points).

The dissimilarities in the factors influencing decisions regarding influenza and COVID-19 were most apparent as percentage differences between shares of participants who expressed their opinion concerning COVID-19 and influenza vaccinations (Figure 5). All but four categories of factors were considered more often in the case of COVID-19 vaccination than influenza vaccination; the exceptions were earlier experience with vaccinations, health status at the time of vaccination, their own personal beliefs concerning vaccinations, and opinions on vaccinations expressed by the clergy and philosophers. Among the factors that gained the most attention regarding COVID-19 vaccination compared to influenza vaccination were the risk of passing on the respective virus to others, the risk of suffering severe complications or death because of the respective infection, and the risk of getting ill because of the respective infection, followed by the availability and cost of vaccination and opinions on the vaccination provided in professional medical information sources.

## 4. Discussion

In recent decades, public health institutions and state authorities worldwide have implemented many different approaches to increase vaccination acceptance levels, both mandatory and elective ones. Unfortunately, in many countries, including Poland, they have become less effective in recent years, most significantly because of organized anti-vaccination movements [37]. This is often interpreted as a failure in the proper delivery of vaccination-related information. Meanwhile, the legal, formal, and economic mechanisms that are supposed to enforce mandatory vaccinations and promote elective ones have become insufficient [38]. It is evident that the worldwide vaccination demise demands urgent investigation in terms of the decision bases accepted by the public, so the most appropriate actions to conserve and improve public health safety can be taken.

Our study group can be considered as resembling, to a significant extent, the general population of medical professionals in Poland. In particular, the majority of the study group consisted of people living in cities (63.13%; >250 K inhabitants), and in Poland, approximately 60% of the general population also lives in cities [39]. The predominant sex in the study group was female, which is consistent with a high level of feminization in the professions of nurses (nearly 100% women) and physicians (around 57% women) in Poland [40], and the healthcare workers in these two professions comprised 74.51% of the study participants. The study group consisted of 42.79% of people who accepted the vaccine against influenza before the last seasonal flu wave, which is fortunate as the results came from both the people already convinced to vaccinate themselves and earlier vaccination skeptics. It is worth mentioning that the share of Polish citizens who finished a basic course of COVID-19 vaccination also recently crossed the threshold of 50% of the whole country’s population [41]. Only in the case of medical professionals and the share of COVID-19-vaccinated workers [33] and, recently, medical students [42] was it reported to be as high as around 90%, as COVID-19 vaccination became obligatory for these groups starting from 1 March 2022 [43].

The flu vaccination rate of the Polish population has been climbing for decades, but it still remains at unsatisfactorily low levels of around 4% for the general population and up to around 8% in the case of medical professions [44]. The study results expose the main underlying factor, which is a low level of fear of getting ill with the flu and its health complications, both for the given person and for other people potentially infected by this person. This renders the given person’s opinions and beliefs concerning vaccinations, and their earlier experiences with vaccinations, as dominating factors that determine their final decision concerning flu vaccination, and this is consistent with findings documented in other studies [6,8,18,22]. It successfully impregnates the person against any information—or observation-based persuasion—by relatives, family, acquaintances, the clergy, philosophers, journals, press, radio, television, the Internet or social media—which were only marginally mentioned by the participants of the study. An exception was made only for professional medical information sources, which were often-declared factors influencing the decision concerning vaccination against influenza. However, these are both available and interesting, but practical only to medical professionals and, thus, cannot be considered a potentially useful means of persuasion for the general public. Unfortunately, this situation leaves little room for hope of improvement in the popularity of flu vaccination in Poland in the coming years.

This study demonstrated a unique situation in the case of COVID-19 vaccination. The obvious limitation here is that all of the study participants were vaccinated against COVID-19, so the study mainly provides information on what factors positively and effectively influenced the attitude toward COVID-19 vaccination. It can be observed that the most commonly declared decision-influencing factors are those associated with fear of the possible threat of COVID-19 to the given person’s own or other people’s health, which was observed by other authors [8,10]. It needed to be strong enough to successfully contradict one’s uncertainty about the vaccine’s efficacy and the fear of its side effects, as stressed by many studies [6,7,8,19,20,24,25,26,27]. This was combined with a lack of experience with the new vaccine that opened people to external communication and observations, which may have made them more open to persuasion. This confirms the importance, raised in the literature, of the information from and the example set by significant others [10], people of authority [7], trendsetters [12,13], and the Internet or social media [28], especially in the case of otherwise indecisive individuals. Together with the low cost (dispensed free) and high availability (administered at the hospital) of the COVID-19 vaccine, all of the abovementioned factors could have swung the decision toward “getting jabbed”—sometimes despite the still strong personal opinions and beliefs concerning vaccinations. It is worth remembering that public health campaigns that are based on a strongly negative—sometimes fearful, or even disgusting—communication appeal to emotions rather than reason, while also resting on solid theoretical foundations [45], have been accepted by societies—initially with some reservations, but in the name of the greater good—and have been widely applied for years and confirmed as effective [46].

The results of the study are in compliance with the results of another study carried out also on Polish population but among unvaccinated patients hospitalized due to COVID-19. It demonstrated that one of the most frequent reasons behind the refusal to receive the vaccine were concerns over the adverse effects and disbelief that the vaccines were sufficiently tested [47], despite the abundant and still increasing knowledge on COVID-19 vaccines and their effects [48]. It is worth stressing, however, that the other most significant reason declared as discouraging people from vaccination was the conviction that the person will not be affected by COVID-19. After succumbing to severe COVID-19, only approximately one-third of them still expressed vaccine hesitancy while the rest of the patients regretted their decision not to receive the vaccine and declared their will to receive it after discharge [47]. 

The results of the study are also consistent with the literature concerning vaccination against COVID-19 in children, which is crucial, considering that it effectively limits the need for hospitalization [49] but even about a quarter of parents declare that they would definitely not have their children vaccinated unless their school required it [50]. It has been established that individually targeted risk-oriented communication is crucial to achieve satisfactory results, but in adults the communicates concentrate on the risk of infecting others while in the case of children it may be more effective to focus on the risk resulting from the SARS-CoV-2 infection for themselves [51].

The vaccination coverage of Poland’s 38 million population calls for urgent improvement, as from several hundred to even 5.4 million influenza cases are registered yearly [52], and the number of COVID-19 cases until mid-June 2022 exceeded 6.0 million, including 116,000 deaths [53].

Based on the available information, it is possible to make a number of generalizations. The study results demonstrated that the decisions concerning vaccinations are significantly based on factors that are difficult to change with rational argumentation, including people’s personal opinions or beliefs concerning vaccinations and their earlier experiences with vaccinations. This explains why it is so difficult to achieve success in the popularization of elective vaccinations required to improve public health, such as vaccination against influenza, which is considered by the public to be a widespread, ordinary, and thus not particularly life-threatening illness. However, the study results suggested that this impregnating effect of opinions, beliefs, and experiences can be surmounted if the vaccine is dispensed free and conveniently while the pathogen is irrationally and emotionally perceived as untamed and potentially life-threatening, as with COVID-19. Consequently, it must be stressed that an effective argumentation for vaccinations cannot rely, as it is predominantly practiced now, solely on flooding the public with positive objective and objective medical information about the benefits of vaccination. However unpopular it may be, for vaccination campaigns to succeed, they need to include strong, subjective, and emotional communication, appealing to negative emotions. They need to exploit the public’s fear of the unknown and stress all of the tangible and personal threats possibly resulting from acquiring a vaccine-preventable infectious disease. Every member of the public must become, sometimes painfully, aware of the seemingly obvious but suppressed fact that these diseases pose a serious threat to the fitness, health, and life of a person, their family, relatives, acquaintances, neighbors, and strangers, causing imminent disturbances in everyday routines and threatening the financial stability and future prospects of all people. A comparison of the effects of vaccination against influenza and COVID-19 demonstrated that only joining the two lines of argumentation may finally allow swinging the decision of the majority of the currently hesitant or objecting public toward vaccination. According to the study results, the power of persuasion by spreading information and setting an example by influential people and modern means of electronic communication is limited during the decision process concerning vaccinations. However small it may seem, the role of this kind of persuasion increases significantly in the case of novel and unfamiliar situations when there are enough collected personal experiences or established personal opinions and beliefs, which confirms the value of perpetual efforts in health education and perseveringly fighting the misinformation on vaccinations.

## 5. Conclusions

The study results demonstrated that decisions concerning vaccinations are typically based on people’s personal opinions or beliefs and their earlier experiences with vaccinations. The impregnating to rational argumentation effect of these factors can be overcome if the vaccine is dispensed free and conveniently, while the pathogen is irrationally and emotionally perceived as untamed and potentially life-threatening. Therefore, the optimal line of argumentation for vaccinations needs to include not only positive objectives and distanced medical information about the benefits of vaccination, but also strong, subjective, and emotional communication, appealing to negative emotions, exploiting the public’s fear of infectious diseases that are perceived as a serious threat to the human fitness, health, and life, disturbing life routines and threatening future prospects, including the financial stability. The study also confirmed the value of perpetual efforts in health education and in fighting the misinformation on vaccinations as the power of persuasion of influential people and modern means of electronic communication is typically limited during the decision process concerning vaccinations but increases significantly in the case of novel and unfamiliar situations.

## Data Availability

Data supporting the reported results are stored by the authors and are available on request.

## References

[1] Leithäuser N., Schneider J., Johann S., Krumke S., Schmidt E., Streicher M. (2021). Quantifying COVID-19-vaccine location strategies for Germany. BMC Health Serv. Res..

[2] Vaishya R., Sibal A., Malani A., Kar S., Kiran S.V., Reddy S., Kamineni S., Reddy S., Reddy P., Reddy P.C. (2021). Symptomatic post-vaccination SARS-CoV-2 infections in healthcare workers—A multicenter cohort study. Diabetes Metab. Syndr. Clin. Res. Rev..

[3] Pascucci D., Nurchis M., Sapienza M., Castrini F., Beccia F., D’ambrosio F. (2021). Evaluation of the effectiveness and safety of the bnt162b2 covid-19 vaccine in the vaccination campaign among the health workers of fondazione policlinico universitario agostino gemelli irccs. Int. J. Environ. Res. Public Health.

[4] Gharpure R., Yi S., Li R., Jacobs Slifka K., Tippins A., Jaffe A. (2021). COVID-19 Vaccine Uptake Among Residents and Staff Members of Assisted Living and Residential Care Communities—Pharmacy Partnership for Long-Term Care Program, December 2020–April 2021. J. Am. Med. Dir. Assoc..

[5] Scardina G., Ceccarelli L., Casigliani V., Mazzilli S., Napoletano M., Padovan M. (2021). Evaluation of flu vaccination coverage among healthcare workers during a 3 years’ study period and attitude towards influenza and potential covid-19 vaccination in the context of the pandemic. Vaccines.

[6] Spinewine A., Pétein C., Evrard P., Vastrade C., Laurent C., Delaere B. (2021). Attitudes towards covid-19 vaccination among hospital staff—understanding what matters to hesitant people. Vaccines.

[7] Kuter B., Browne S., Momplaisir F., Feemster K., Shen A., Green-McKenzie J. (2021). Perspectives on the receipt of a COVID-19 vaccine: A survey of employees in two large hospitals in Philadelphia. Vaccine.

[8] Maltezou H., Pavli A., Dedoukou X., Georgakopoulou T., Raftopoulos V., Drositis I. (2021). Determinants of intention to get vaccinated against COVID-19 among healthcare personnel in hospitals in Greece. Infect. Dis. Health.

[9] Nohl A., Abdallah H., Weichert V., Zeiger S., Ohmann T., Dudda M. (2021). A local survey of COVID-19: Vaccine potential acceptance rate among personnel in a level 1 trauma center without severe COVID-19 cases. Healthcare.

[10] Huynh G., Tran T., Nguyen H., Pham L. (2021). COVID-19 vaccination intention among healthcare workers in Vietnam. Asian Pac. J. Trop. Med..

[11] Sirikalyanpaiboon M., Ousirimaneechai K., Phannajit J., Pitisuttithum P., Jantarabenjakul W., Chaiteerakij R. (2021). COVID-19 vaccine acceptance, hesitancy, and determinants among physicians in a university-based teaching hospital in Thailand. BMC Infect. Dis..

[12] Myers V., Saban M., Ben-Shetrit S., Wilf-Miron R. (2021). Uptake of COVID-19 vaccination among general hospital staff in Israel. Infect. Control Hosp. Epidemiol..

[13] Fotiadis K., Dadouli K., Avakian I., Bogogiannidou Z., Mouchtouri V., Gogosis K. (2021). Factors associated with healthcare workers’ (HCWs) acceptance of COVID-19 vaccinations and indications of a role model towards population vaccinations from a cross-sectional survey in Greece, May 2021. Int. J. Environ. Res. Public Health.

[14] Forgeschi G., Cavallo G., Lorini C., Balboni F., Sequi F., Bonaccorsi G. (2021). Investigating adherence to COVID-19 vaccination and serum antibody concentration among hospital workers—The experience of an italian private hospital. Vaccines.

[15] Drobnik J., Suslo R., Pobrotyn P., Fabich E., Magiera V., Diakowska D., Uchmanowicz I. (2021). COVID-19 among healthcare workers in the University Clinical Hospital in Wroclaw, Poland. Int. J. Environ. Res. Public Health.

[16] Gostin L., Salmon D., Larson H. (2021). Mandating COVID-19 vaccines. JAMA.

[17] Olick R., Shaw J., Yang Y. (2021). Ethical Issues in Mandating COVID-19 Vaccination for Health Care Personnel. Mayo Clin. Proc..

[18] Mena G., Blanco B., Casas I., Huertas A., Sánchez M., Auñón M. (2021). Attitudes of Spanish hospital staff towards COVID-19 vaccination and vaccination rates. PLoS ONE.

[19] Vignier N., Brureau K., Granier S., Breton J., Michaud C., Gaillet M. (2021). Attitudes towards the COVID-19 vaccine and willingness to get vaccinated among healthcare workers in french guiana: The influence of geographical origin. Vaccines.

[20] Kara Esen B., Can G., Pirdal B., Aydin S., Ozdil A., Balkan I. (2021). Covid-19 vaccine hesitancy in healthcare personnel: A university hospital experience. Vaccines.

[21] Dugani S., Geyer H., Maniaci M., Fischer K., Croghan I., Coons T. (2021). Hospitalist perspectives on barriers to recommend and potential benefit of the COVID-19 vaccine. Hosp. Pract..

[22] El Kefi H., Kefi K., Krir M., Brahim C., Baatout A., Bouzouita I. (2021). Acceptability of COVID-19 vaccine: A cross-sectional study in a Tunisian general hospital. Pan Afr. Med. J..

[23] Robbins T., Kyrou I., Clark C., Sharma K., Laird S., Berry L. (2021). Healthcare staff perceptions following inoculation with the bnt162b2 mrna covid-19 vaccine at university hospitals coventry & warwickshire nhs trust. Int. J. Environ. Res. Public Health.

[24] Bauernfeind S., Hitzenbichler F., Huppertz G., Zeman F., Koller M., Schmidt B. (2021). Brief report: Attitudes towards COVID-19 vaccination among hospital employees in a tertiary care university hospital in Germany in December 2020. Infection.

[25] Abuown A., Ellis T., Miller J., Davidson R., Kachwala Q., Medeiros M. (2021). COVID-19 vaccination intent among London healthcare workers. Occup. Med..

[26] Mori Y., Miyatake N., Suzuki H., Okada S., Tanimoto K. (2021). A text mining-based survey of pre-impressions of medical staff toward COVID-19 vaccination in a designated medical institution for class ii infectious diseases. Vaccines.

[27] Martin C., Marshall C., Patel P., Goss C., Jenkins D., Ellwood C. (2021). SARS-CoV-2 vaccine uptake in a multi-ethnic UK healthcare workforce: A cross-sectional study. PLoS Med..

[28] Huang X., Yu M., Fu G., Lan G., Li L., Yang J. (2021). Willingness to Receive COVID-19 Vaccination among People Living with HIV and AIDS in China: Nationwide Cross-sectional Online Survey. JMIR Public Health Surveill..

[29] Kalbhenn J., Hammer T., Hug M., Dürschmied D., Wenz F., Steinmann D. (2021). Subjective well-being and ability to work of hospital staff after SARS-CoV2 immunization with the mRNA vaccine BNT162b2. Dtsch. Med. Wochenschr..

[30] Rozporządzenie Rady Ministrów z Dnia 14 Grudnia 2021 r. Zmieniające Rozporządzenie w Sprawie Ustanowienia Określonych Ograniczeń, Nakazów i Zakazów w Związku z Wystąpieniem Stanu Epidemii (Dz.U. 2021 poz. 2311). https://isap.sejm.gov.pl/isap.nsf/download.xsp/WDU20210002311/O/D20212311.pdf.

[31] Pfizer and Biontech Uzyskują Dopuszczenie do Obrotu w Unii Europejskiej Szczepionki Przeciw COVID-19. https://www.pfizer.pl/files/12_21_Uzyskanie-dopuszczenia-do-obrotu-w-UE.pdf.

[32] Szarowska A., Zaczyński A., Szymański P., Borawska B., Szarek I., Szoszkiewicz I., Butkiewicz S., Szydlarska D., Gil R., Śliwczyński A. (2021). The Central Clinical Hospital of the Ministry of Internal Affairs and Administration Program Coordination Group OBO. Initiation of the COVID-19 vaccination program in Poland: Vaccination of the patient “zero” and first experience from the Central Clinical Hospital of the Ministry of Internal Affairs and Administration. Pol. Arch. Intern. Med..

[33] Naczelna Izba Lekarska (2021). Szczepienia Lekarzy i Lekarzy Dentystów Przeciwko COVID-19 w Liczbach. https://nil.org.pl/aktualnosci/5359-szczepienia-lekarzy-i-lekarzy-dentystow-p-covid-19-w-liczbach.

[34] Dąbek A. (2021). Ile Lekarzy i Pielęgniarek w Polsce Zostało Zaszczepionych? Medonet.pl. https://www.medonet.pl/porozmawiajmyoszczepionce,szczepienia-przeciw-covid-19--ilu-jest-zaszczepionych-lekarzy-w-polsce,artykul,32314696.html.

[35] Byhoff E., Paulus J., Guardado R., Zubiago J., Wurcel A. (2021). Healthcare workers’ perspectives on coronavirus testing availability: A cross sectional survey. BMC Health Serv. Res..

[36] Matejuk T. (2021). Koronawirus we Wrocławiu, na Dolnym Śląsku, w Polsce. Wroclife.pl. https://wroclife.pl/nasze-miasto/koronawirus-wroclaw-2020/koronawirus-dolny-slask-13-grudnia-2021/.

[37] Paplicki M., Susło R., Najjar N., Ciesielski P., Augustyn J., Drobnik J. (2018). Conflict of individual freedom and community health safety: Legal conditions on mandatory vaccinations and changes in the judicial approach in the case of avoidance. Fam. Med. Prim. Care Rev..

[38] Paplicki M., Susło R., Benedikt A., Drobnik J. (2020). Effectively enforcing mandatory vaccination in Poland and worldwide. Fam. Med. Prim. Care Rev..

[39] Central Statistical Office (2022). Population: Size and Structure and Vital Statistics in Poland by Territorial Division in 2019.

[40] Central Statistical Office (2022). Statistical Information and Elaborations—Health and Health Care in 2020, Medical Personnel.

[41] Polska na 23 (2022). Miejscu w UE pod Względem Odsetka Zaszczepionych Osób. 300gospodarka.pl. https://300gospodarka.pl/wykres-dnia/polska-miejsce-w-europie-szczepienia-covid-19.

[42] (2022). Która z Polskich Uczelni ma Najwyższy Odsetek Zaszczepionych Studentów? Medexpress.pl. https://www.medexpress.pl/ktora-z-polskich-uczelni-ma-najwyzszy-odsetek-zaszczepionych-studentow/83748.

[43] Rozporządzenie Ministra Zdrowia z Dnia 22 Grudnia 2021 r. Zmieniające Rozporządzenie w Sprawie Ogłoszenia na Obszarze Rzeczypospolitej Polskiej Stanu Epidemii (Dz. U. 2021 poz. 2398). https://isap.sejm.gov.pl/isap.nsf/download.xsp/WDU20210002398/O/D20212398.pdf.

[44] Susło R., Pobrotyn P., Brydak L., Rypicz Ł., Grata-Borkowska U., Drobnik J. (2021). Seasonal Influenza and Low Flu Vaccination Coverage as Important Factors Modifying the Costs and Availability of Hospital Services in Poland: A Retrospective Comparative Study. Int. J. Environ. Res. Public Health.

[45] Peters G.-J., Ruiter R., Kok G. (2012). Threatening communication: A critical re-analysis and a revised meta-analytic test of fear appeal theory. Health Psychol. Rev..

[46] Halkjelsvik T. (2014). Do disgusting and fearful anti-smoking advertisements increase or decrease support for tobacco control policies?. Int. J. Drug Policy.

[47] Zarębska-Michaluk D., Rzymski P., Moniuszko-Malinowska A., Brzdęk M., Martonik D., Rorat M., Wielgat J., Kłos K., Musierowicz W., Wasilewski P. (2022). Does Hospitalization Change the Perception of COVID-19 Vaccines among Unvaccinated Patients?. Vaccines.

[48] Torres-Estrella C.U., Reyes-Montes M.d.R., Duarte-Escalante E., Sierra Martínez M., Frías-De-León M.G., Acosta-Altamirano G. (2022). Vaccines Against COVID-19: A Review. Vaccines.

[49] Araujo da Silva A.R., de Carvalho B.R.R., Esteves M.d.M., Teixeira C.H., Souza C.V. (2022). The Role of COVID-19 Vaccinal Status in Admitted Children during OMICRON Variant Circulation in Rio de Janeiro, City—Preliminary Report. Vaccines.

[50] McElfish P.A., Willis D.E., Shah S.K., Reece S., Andersen J.A., Schootman M., Richard-Davis G., Selig J.P., Warmack T.S. (2022). Parents’ and Guardians’ Intentions to Vaccinate Children against COVID-19. Vaccines.

[51] Van Hoecke A.L., Sanders J.G. (2022). An Online Experiment of NHS Information Framing on Mothers’ Vaccination Intention of Children against COVID-19. Vaccines.

[52] PZH-NIZP Zachorowania i podejrzenia zachorowań na grypę w Polsce. Państwowy Zakład Higieny—Narodowy Instytut Zdrowia Publicznego. http://wwwold.pzh.gov.pl/oldpage/epimeld/grypa/index.htm.

[53] Koronawirus w Polsce (SARS-CoV-2). Koronawirusunas.pl. https://koronawirusunas.pl/.

